# 584. Implementation and Evaluation of an Interactive Infectious Diseases Online Curriculum for Trainees on ID electives

**DOI:** 10.1093/ofid/ofae631.179

**Published:** 2025-01-29

**Authors:** Gayathri Krishnan, Caline Mattar, Nigar Kirmani, Steven Lawrence

**Affiliations:** Washington University School of Medicine, St. Louis, MO; Washington University School of Medicine, St. Louis, MO; Washington University School Of Medicine, St Louis, Missouri; Washington University in Saint Louis School of Medicine, Saint Louis, MO

## Abstract

**Background:**

Infectious Diseases Online Curriculum (IDOC) is an online asynchronous interactive educational resource for trainees on ID electives to augment their ID knowledge and complement their clinical training. The curriculum mapping with details of modules and methods of assessment are elaborated in TABLE 1. A program evaluation of the IDOC with the aim to analyze program outcomes was done in 2023.

**Table 1:**
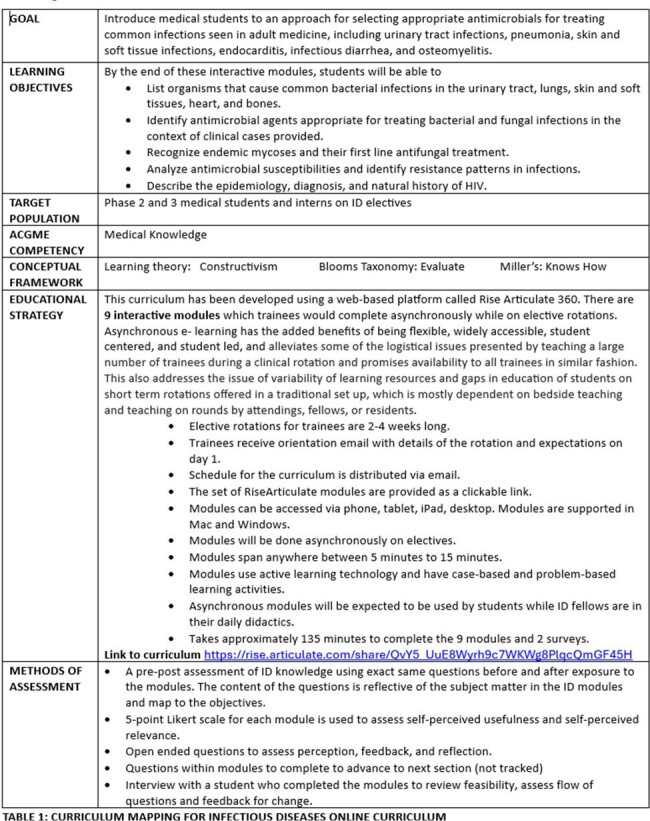
Curriculum mapping including goals, objectives, conceptual framework, educational strategies and assessments for Infectious Diseases Online Curriculum

**Methods:**

IDOC was piloted with medical students and residents from August 2022 to December 2022 and assesses Kirkpatrick level 2 learning. The primary assessment was of Program Outcome, set up using a Pragmatism paradigm with Mixed Methods research methodology to assess quantitative and qualitative data. Following IRB approval, data collection was conducted through web-based surveys (Qualtrics). Each surveyor was asked to assign a unique alphanumeric number to their pre and post survey so these could be used for comparison. The primary outcome assessed was change in knowledge. Each question in the pre- and post-assessment was awarded 1 for correct answer and 0 for incorrect answer. A paired sample t test was used to compare pre and post cumulative scores and analyzed using SPSS v26.

**Table 2:**
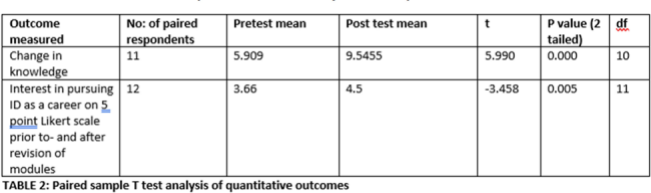
Paired sample T test analysis of quantitative outcomes

**Results:**

During the pilot phase 16 pre- and 20 post assessment surveys were received (8 phase-3 students, 4 phase-2, 3 phase-1, 3 interns, 2 with no level of training). 11 respondents had allotted the same alphanumeric number to both pre- and post-surveys and these were used for comparative analyses of knowledge change. Interest in pursuing ID as a career prior to and after revision of modules was sought on a 5-point Likert scale and received 12 responses. Paired t test showed significant improvement in knowledge and increase in interest of trainees to pursue ID as a career (TABLE 2). Majority of trainees also rated the modules to be extremely clear and relevant to clinical practice (TABLE 3). Qualitative feedback was overwhelmingly positive (TABLE 4).

**Table 3:**
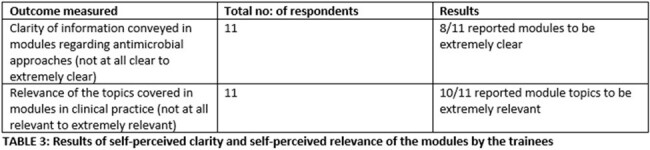
Results of self-perceived clarity and self perceived relevance of the modules by the trainees

**Conclusion:**

IDOC functions as a novel, accessible, quick, engaging, and effective way to improve ID related knowledge in trainees and is an adjunct to their clinical elective experience. Although this was a small study, the results are significant and transferable, and IDOC can be disseminated to smaller training programs where educators and ID learning resources are not available.

**Table 4:**
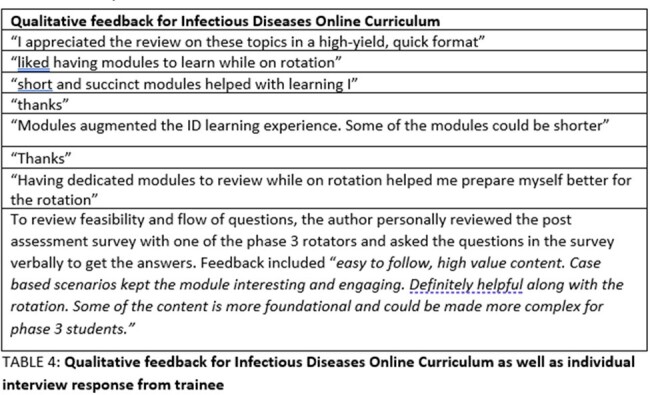
Qualitative feedback for Infectious Diseases Online Curriculum as well as individual interview response from trainee

**Disclosures:**

**All Authors**: No reported disclosures

